# Prediction of present and future distribution areas of *Juniperus drupacea* Labill and determination of ethnobotany properties in Antalya Province, Türkiye

**DOI:** 10.1515/biol-2022-0883

**Published:** 2024-06-18

**Authors:** Guliz Turkmenoglu, Ayse Gul Sarikaya, Almira Uzun, Huseyin Fakir

**Affiliations:** Akseki Vocational School, Alanya Alaaddin Keykubat University, Antalya, Turkey; Faculty of Forestry, Bursa Technical University, Bursa, Turkey; Faculty of Forestry, Isparta University of Applied Sciences, Isparta, Turkey

**Keywords:** Andiz (*Juniperus drupacea*), Andiz molasses, climate change, ethnobotany, maximum entropy

## Abstract

Ethnobotanical studies revealed the experience and knowledge of people who learned the therapeutic virtues of plants through trials and errors and transferred their knowledge to the next generations. This study determined the ethnobotanical use of *Juniperus drupacea* (Andiz) in the Antalya province and the current and future potential distribution areas of *J. drupacea* in Türkiye during 2041–2060 and 2081–2100 according to the SSP2-4.5 and SSP5-8.5 scenarios and based on the IPSL-CM6A-LR climate change model. The very suitable areas encompassed 22379.7 km^2^. However, when the SSP2-4.5 scenario was considered, the areas most suitable for *J. drupacea* comprised 6215.892 km^2^ for 2041–2060 and 378.318 km^2^ for 2081–2100. Based on the SSP5-8.5 scenario, the area most suitable for *J. drupacea* was 979.082 km^2^ for 2041–2060. However, no suitable areas were identified with the SSP5-8.5 scenario for 2081–2100. Considering the models for the future estimated distribution areas of *J. drupacea*, serious contractions endangering the species are predicted in its distribution areas. Therefore, scientific research should focus on identifying *J. drupacea* populations and genotypes that demonstrate resilience to future drought conditions resulting from climate change. This endeavor is crucial as it holds significant ecological and economic values.

## 1 Introduction

Humans have always benefited from nature to meet their food, clothing, cooking, and fuel needs. The information obtained through trial and error has been developed and carried to the present day, and ethnobotanical studies have gained importance [1–4]. In recent years, technological development, urban transitions, modern life, and the relative loss of traditions and customs caused a decrease in orally transmitted information about plants. In this respect, ethnobotanical studies are valuable for recording “humanity’s knowledge of plants” [3].

Türkiye is rich in flora and plant diversity, and ethnobotanical studies are still conducted by many researchers in different regions of the country; however, the actual use of the plants identified by such studies remains poorly known. Recently, the demand for forest and natural resources has increased. This increase is essential in meeting commercial gains and local needs, especially for forest villagers.

Cupressaceae is a monoic or dioecious family. Its members are evergreen small trees or shrubs, resinous, distinctively fragrant, and highly branched. Some leaves are needle-shaped, while others are flakes [5,6]. *Juniperus drupacea* Labill, belonging to the family Cupressaceae, is a wingless tree with thin trunk shells and seeds. It mostly grows on the Mediterranean coast and back-Mediterranean forests at 600–1,750 m of altitude [5,7]. It is naturally distributed in Türkiye and has socio-economic and ecological contributions. Different parts of the tree are used as folk medicine and food. It is used to treat respiratory and urinary tract infections, diarrhea, and abdominal pain. Additionally, the molasses obtained from its fruits is consumed as food; its local consumption has spread throughout Türkiye in recent years as food and food support [5].

*J. drupacea* is endangered because of natural regeneration, logging, forbidden grazing, illegal collection for ethnobotanical use, and climate change. The natural regeneration of the species is difficult due to slow growth, low seed viability, and germination problems [8]. Additionally, the decrease in juniper stands destroys nutrient-rich soils well suited for seed growing under denser tree covers [9,10]. This situation threatens the survival of juniper species. Therefore, taking measures to protect existing juniper stands and ensure their sustainability is necessary [11].

This study aimed to determine the ethnobotanical use of *J. drupacea* in the Antalya region. Determining the usage areas of plants and their local names is essential for transferring human heritage to future generations. Additionally, with technological development and urban transitions, the loss of ethnobotanical information becomes problematic, making the regular recording of such information crucial.

The second part of the study aimed to determine the potential distribution areas of *J. drupacea* over time based on climate change scenarios using maximum entropy (MaxEnt). Climate change increases the danger of plant species extinction by negatively affecting their biodiversity and geographical distribution [12,13]. Reintroducing and rehabilitating threatened species in terrestrial ecosystems requires detailed knowledge of their potential distribution range. Global scientific studies and observations have determined that plants migrate to high altitudes due to climate change, which will continue in the next 100 years [14–16]. MaxEnt, an algorithm used to model the appropriate geographical distribution of species based on the species distribution model, was used in this study [17]. An evaluation based on machine learning was made to determine future strategies for the conservation and sustainability of *J. drupacea.*

## 2 Materials and methods

### 2.1 Determining ethnobotany properties

A questionnaire of 20 questions was prepared, and interviews were conducted with the local people to determine the ethnobotanical characteristics of *J. drupacea* in the Kuyucak district of Antalya province. *J. drupacea* is widespread throughout the Mediterranean region of Türkiye, and Kuyucak district was chosen as the study area because it produces Andiz molasses densely.

Sixty-five people were interviewed in the region. The questionnaire included the following questions, among others: (Question 6) What is the situation on plant use that grows naturally in the region? (Question 8) For what purpose do you use the Andiz tree? (Question 10) What is taken into consideration when collecting the Andiz tree from nature? (Question 11) How are the collected plants stored? Moreover, general research was conducted on Questions 8–14. For multiple-choice questions, each option was evaluated as a % in itself.

Participants were allowed to answer the questionnaire alone, at their own free will, without external influence. The following equation was used to determine the sample size [18]:

\[n=\frac{{Z}^{2}{Npq}}{{{ND}}^{2}+{Z}^{2}{pq}},]\]

where *n* is the sample size, *Z* is the confidence coefficient (*Z* = 1.96 for 95% confidence interval), *N* is the main mass size, *p* and *q* are the probability of finding the desired size in the population (0.5), and *D* is the accepted sampling error (10%).

Using this equation, the sample size was found to be 50. The survey of 65 people was considered, including the margin of error. Questionnaires were evaluated by converting the numerical values of the answered choices into percentages in frequency tables. Statistical Package for Social Science 25.0 was used for the analysis.

### 2.2 Prediction of present and future potential distribution areas of *J. drupacea*

**Figure 1:**
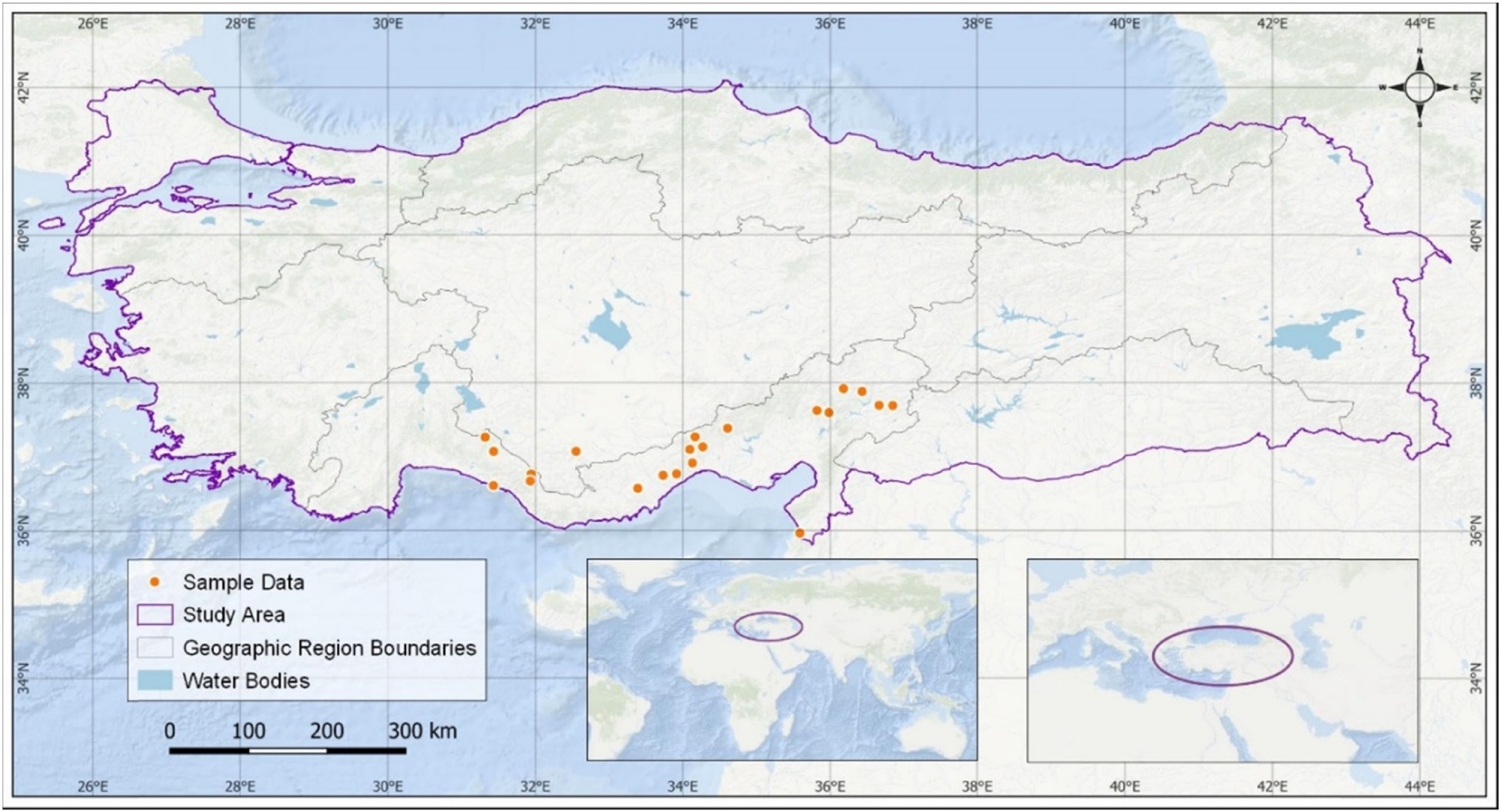
Input data for the MaxEnt area of *J. drupacea*.

MaxEnt 3.4.1, which produces a species distribution model, was used to determine the potential distribution areas of *J. drupacea* according to climate change scenarios. The existing data of the species (sample points), environmental variables (bioclimatic data), and future climate change scenarios are required to obtain results from the MaxEnt algorithm. Sample spots of *J. drupacea* were obtained from available literature and field observations (Table 1) [19–22]. The coordinates of these points were marked in the WGS84 coordinate system in the current version of the QGIS 3.22 program utilizing Google Earth Satellite 5 m resolution base maps as the base data (Figure 1) [23].

**Table 1:** Attribute information of *J. drupacea* Labill

*J. drupacea*	*X*	*Y*	Province	District	Avg. sol. rad.	Avg. temp.	Avg. wind	Avg. prec.	Elev.
1	35.006	37.382	Adana	Karaisalı	2.06	11.79	2.06	52.71	1,001
2	31.83	37.07	Antalya	Akseki	2.33	10.50	2.33	57.13	1,500
3	31.715	37.261	Antalya	Akseki	2.17	10.77	2.17	55.44	1,201
4	32.326	36.668	Antalya	Alanya	2.63	9.05	2.63	60.70	1,767
5	31.828	36.607	Antalya	Alanya	2.36	18.84	2.36	86.16	41
6	32.338	36.764	Antalya	Gündoğmuş	2.62	8.15	2.62	59.47	1,836
7	35.989	35.963	Hatay	Yayladağı	3.28	13.69	3.28	96.12	885
8	37.244	37.69	Kahramanmaraş	Çağlayancerit	2.21	10.34	2.21	46.84	1,386
9	36.381	37.595	Kahramanmaraş	Andırın	2.07	10.98	2.07	58.02	1,243
10	37.06	37.693	Kahramanmaraş	Dulkadiroğlu	2.17	10.17	2.17	47.54	1,384
11	36.578	37.918	Kahramanmaraş	Onikişubat	2.13	9.48	2.13	51.50	1,424
12	36.83	37.88	Kahramanmaraş	Onikişubat	2.07	11.13	2.07	50.80	1,126
13	32.955	37.071	Karaman	Karaman M.	2.26	9.71	2.26	43.07	1,460
14	34.566	37.265	Mersin	Çamlıyayla	2.39	7.53	2.39	42.84	1,660
15	34.67	37.13	Mersin	Çamlıyayla	2.31	13.29	2.31	54.01	938
16	34.134	36.746	Mersin	Erdemli	2.67	11.06	2.67	51.34	1,399
17	34.316	36.767	Mersin	Erdemli	2.62	13.46	2.62	57.49	793
18	33.791	36.569	Mersin	Silifke	2.63	10.63	2.63	51.88	1,504
19	34.495	37.096	Mersin	Toroslar	2.38	11.28	2.38	46.56	1,289
20	34.53	36.913	Mersin	Toroslar	2.48	15.07	2.48	59.78	687
21	36.219	37.622	Osmaniye	Kadirli	1.97	10.90	1.97	58.67	1,253

The bioclimatic data from the WorldClim [24] data-sharing site comprise 19 bioclimatic variables produced from the lowest and highest temperature averages and annual precipitation averages (Table 2) to find the current potential distribution areas. Descriptive data, such as elevation, aspect, slope, temperature, precipitation, humidity, and insolation, for sample points were collected using maps from the database [24]. Predictive modeling of the current and future potential distribution areas relied specifically on the WorldClim database, particularly version 2.1 [24]. This database includes monthly climate data from 1970 to 2000, encompassing minimum, average, and maximum temperatures, precipitation, solar radiation, wind speed, water vapor pressure, and total precipitation.

**Table 2:** Bioclimatic variables (WorldClim [24])

BIO1 = Annual mean temperature
BIO2 = Mean diurnal range (mean of monthly (max temp − min temp))
BIO3 = Isothermality (BIO2/BIO7) (×100)
BIO4 = Temperature seasonality (standard deviation × 100)
BIO5 = Max temperature of warmest month
BIO6 = Min temperature of coldest month
BIO7 = Temperature annual range (BIO5–BIO6)
BIO8 = Mean temperature of wettest quarter
BIO9 = Mean temperature of driest quarter
BIO10 = Mean temperature of warmest quarter
BIO11 = Mean temperature of coldest quarter
BIO12 = Annual precipitation
BIO13 = Precipitation of wettest month
BIO14 = Precipitation of driest month
BIO15 = Precipitation seasonality (coefficient of variation)
BIO16 = Precipitation of wettest quarter
BIO17 = Precipitation of driest quarter
BIO18 = Precipitation of warmest quarter
BIO19 = Precipitation of coldest quarter

Bioclimatic variables, with a spatial resolution of 2.5 min (approximately 20 km²), were utilized to determine the current distribution and were derived from data observed in WorldClim version 2.1. Detailed information regarding these specific variables is provided in Table 2. The IPSL-CM6A-LR climate change model, which is the latest version of the Institut Pierre-Simon Laplace (IPSL) climate model and also includes the carbon cycle, was used to extract the future distribution areas of *J. drupacea* [25]. According to the SSP2-4.5 and SSP5-8.5 scenarios, the potential distribution areas of the species in the 2041–2060 (∼2050) and 2081–2100 (∼2090) time intervals were modeled and presented with maps and tables. Based on the model results, the loss and gain values in the species’ distribution areas were deduced from the intersection of the current and future potential distribution areas.

**Figure 2:**
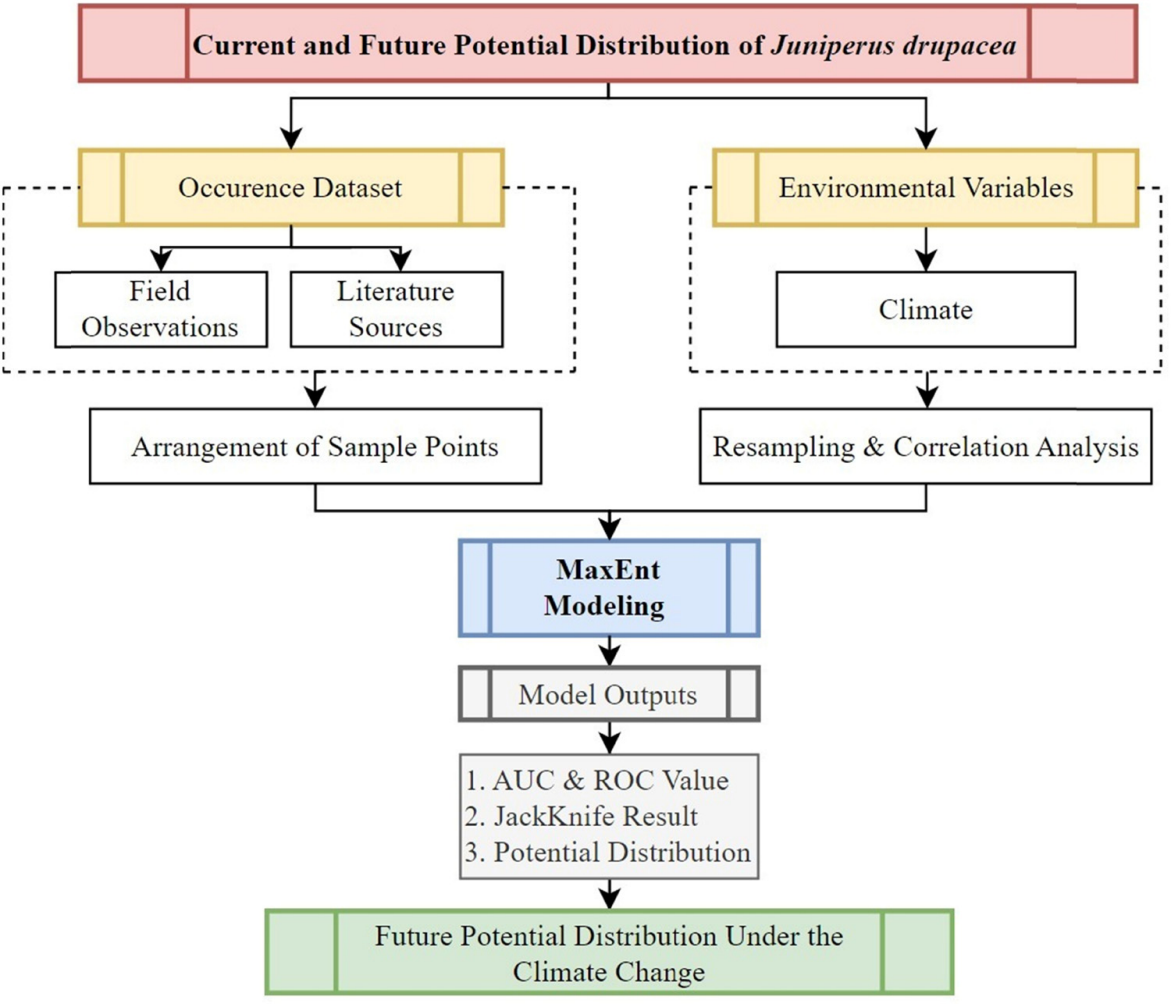
Flowchart illustrating the methodology employed for MaxEnt modeling and forecasting the future potential distribution of *J. drupacea* under climate change scenarios.

The overall methodological flowchart for the MaxEnt modeling of *J. drupacea* is presented in Figure 2. The flowchart comprises four main steps: (1) preparing and pre-processing the environmental variables, (2) assessing crucial factors and predicting potential distribution areas using MaxEnt models under various climate scenarios, (3) validating the distribution through field investigations and statistical methods, and (4) analyzing the characteristics of the range shift for *J. drupacea.*

## 3 Results and discussion

### 3.1 Ethnobotany properties

The survey participants included 56.9% of women and 43.1% of men, among which 69.7% were married. The average age of participants was 60 years and above. About 26.2% of the participants were primary school-secondary education graduates, 52.9% were unemployed/housewives, 34.4% were self-employed, and 12.6% were public sector workers (Table 3).

**Table 3:** Demographic characteristics of survey participants

	Number	Percentage
**Sex**		
Female	37	56.9
Male	28	43.1
Total	65	100
**Marital status**		
Single	20	30.3
Married	45	69.7
Total	65	100
**Age**		
18–30	5	7.7
31–40	11	17
41–50	14	21.5
51–60	10	15.4
>60	25	38.4
Total	65	100
**Education status**		
İlliterate	5	7.7
Primary school	17	26.2
Secondary school	17	26.2
High school	8	12.3
University/undergraduate	15	23
Master	2	3.1
PhD	1	1.5
Total	65	100
**Profession**		
Housewife-not working	34	52.9
Self-employment	22	34.4
Government official	9	12.6
Total	119	100

Around 98.5% of the participants benefited from the plants that grow naturally in the region and used *J. drupacea*. According to the research results, 98.5% of the local people benefited from plants for health and treatment, 88.1% for food/spices, 1.5% for cosmetic and aesthetic purposes, and 1.5% for pleasure. About 87.7% of the participants obtained the plants by collecting them from nature.

Regarding which parts of the plant they most benefit from, 98.5% mentioned the cones, 3.1% the flowers, and 1.5% the leaves. When asked about the collection period, 23.1% answered October–November and September–October, 18.5% September–November, 13.8% October, 1.5% October–November, and 6.2% October–December. 9.2% said they did not collect the plant. About the Andiz they collected, 95.4% stated they preserved it as molasses, and 6.2% dried the cones, flowers, and leaves. When asked about their consumption time, 84.6% said as they get sick, 35.4% as needed, 20% when needed, and 1.5% every day. When collecting *J. drupacea* from nature, 87.7% pay attention to the mature cones, 52.3% to collect in the right season, 23.1% to the plants’ health, 16.9% to the cleanliness and hygiene of the collection area, 4.6% to the completeness of all the plant’s organs. When asked if they had heard about the potential side effects of using the plant, 56.9% said no and 43.1% said they had heard of side effects but did not see any. Among the factors influencing people’s consumption, 83.1% were from family, 72.3% from people around, 6.2% from printed media, 3.1% from books/magazines, 1.5% from audio-visual media, and 1.5% from advertisements. Finally, when asked about the way they consume the plant, 78% said as molasses when they get sick, one spoon a day, 11% as molasses two spoons a week, and 11% when needed. 96% of the participants stated that they obtained molasses by boiling the cones of the Andiz tree and 4% stated that they bought it ready-made. To make molasses, Andiz cones are collected from nature and passed through a pine cone crushing machine to obtain flour. The flour is kept in water for 48 h and filtered through a sieve before boiling. Boiling continues until the flour turns into molasses. The formed pulp is taken out during boiling.

About 44% of the participants reported selling the products they obtained from the Andiz tree and sending the molasses to companies in metropolitan cities. 45% of them sell it in local markets, 7% do not sell it but collect enough molasses for their own family’s consumption, and 4% stated that they do not sell it but transfer it ready-made and buy it from the markets. About the way they consume the molasses obtained from the Andiz, 41% answered a spoonful a day to treat colds, 40% said a spoonful twice a week as an immune booster, and 12% said one spoonful a day to treat fatigue, 4% answered one spoonful a day as an appetizer, and 3% consume one spoonful every other day to treat stomach discomfort, sore throat, runny nose, and loss of appetite. Molasses has reached a central position today with the development of production technologies and the increasing interest in nutrient-rich natural products [26].

Andiz tree fruits contain a hard seed, from which Andiz rosary is made. Andiz molasses is made from the outer shells of the Andiz cones. In the villages of the Taurus Mountains, “Andiz Molasses” is traditionally obtained by boiling young cones with water. The taste of this molasses is slightly bitter, and its production is limited since it is laborious. Andiz molasses can regulate the amount of sugar in the blood and is good for anemia; it is used to treat bronchitis, cough, mouth sores, tuberculosis, kidney inflammation, psoriasis, nausea, and hemorrhoids and is beneficial for the lungs and liver. Andiz molasses is especially rich in minerals; the potassium, calcium, phosphorus, magnesium, and sodium content of Andiz molasses produced by traditional methods is quite high, especially for grape, carob, and fig molasses [27]. The results of our survey indicate that molasses obtained from the fruits of the Andiz tree is used extensively by the local people to treat various diseases.

### 3.2 Prediction of potential distribution areas of *J. drupacea*

**Figure 3:**
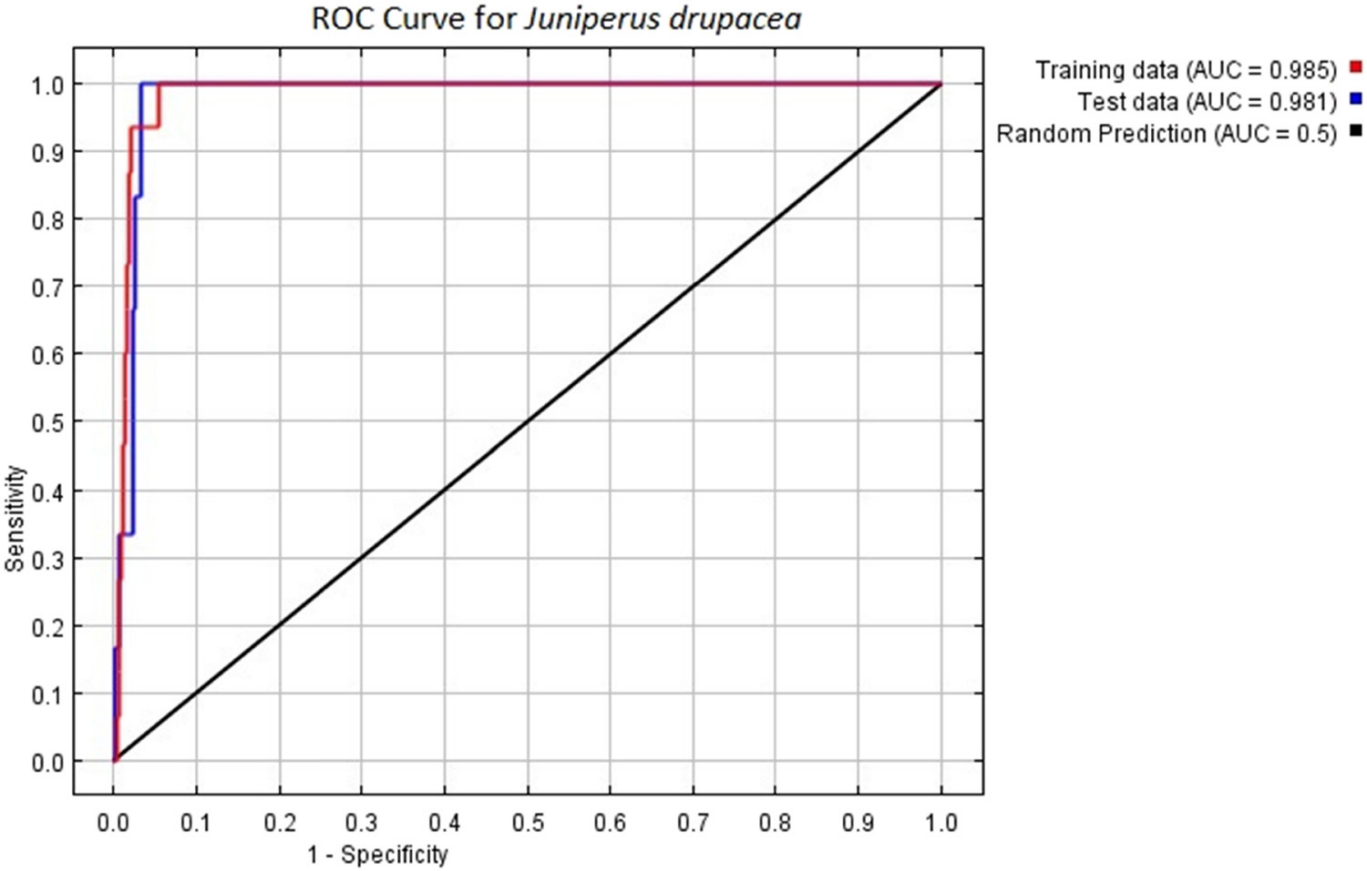
ROC curve for *J. drupacea*.

The success of MaxEnt models was tested with a receiver operating characteristic (ROC). The model’s accuracy increases as the area under the ROC curve (AUC) value approaches 1. If the AUC exceeds 0.5, the model performs better than random prediction [28–32]. The AUC value of 0.985 in the model output indicates that the model is very sensitive and accurate (Figure 3).

**Figure 4:**
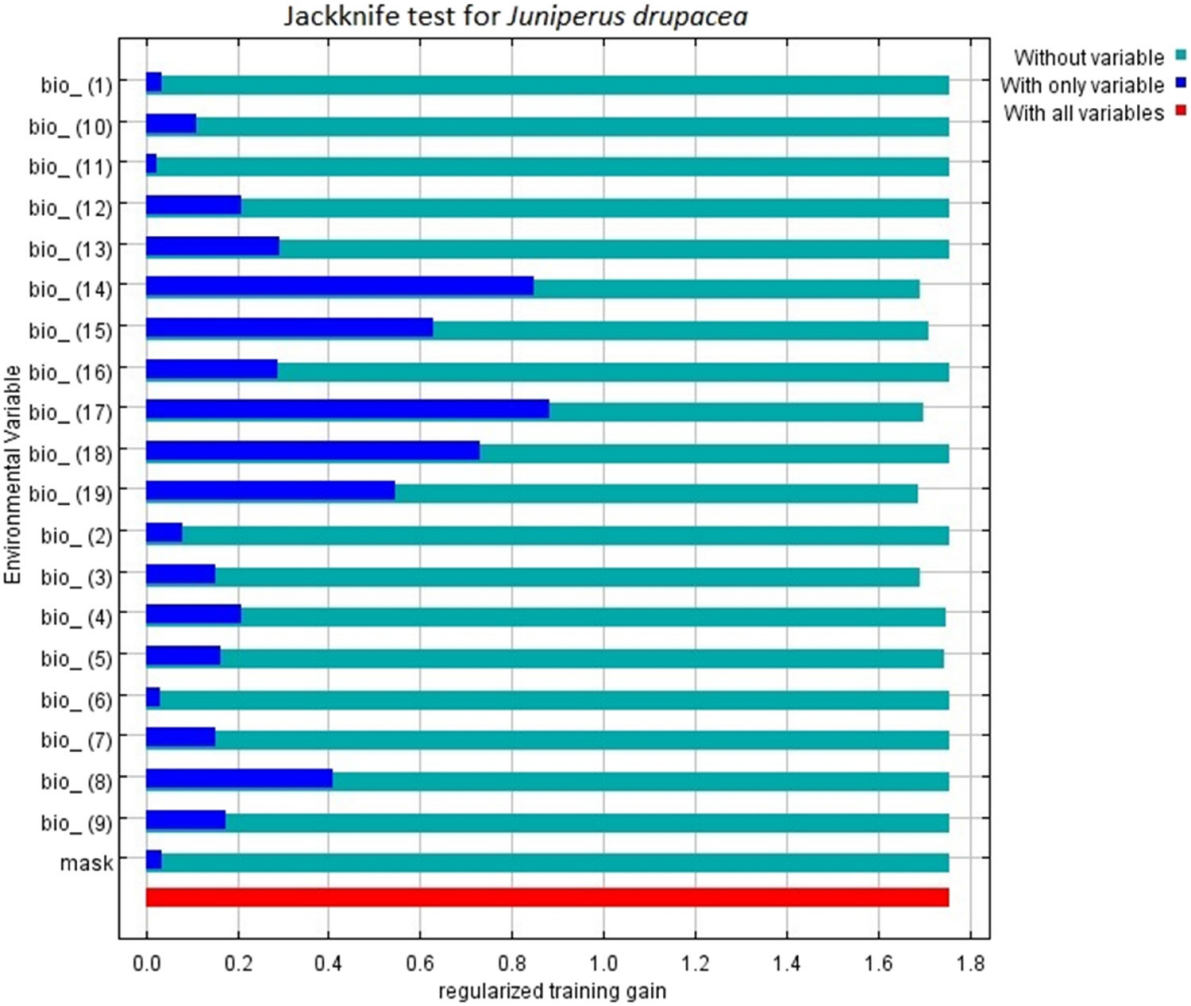
Jackknife test for *J. drupacea*.

The MaxEnt model also offers the Jackknife test option, which presents the importance levels of the variables during the process. The Jackknife test results for *J. drupacea* indicate that the most important variables are related to precipitation, including the Precipitation of Driest Quarter (BIO17), Precipitation of Driest Month (BIO14), Precipitation of Warmest Quarter (BIO18), and Precipitation Seasonality (BIO15) (Figure 4).

**Figure 5:**
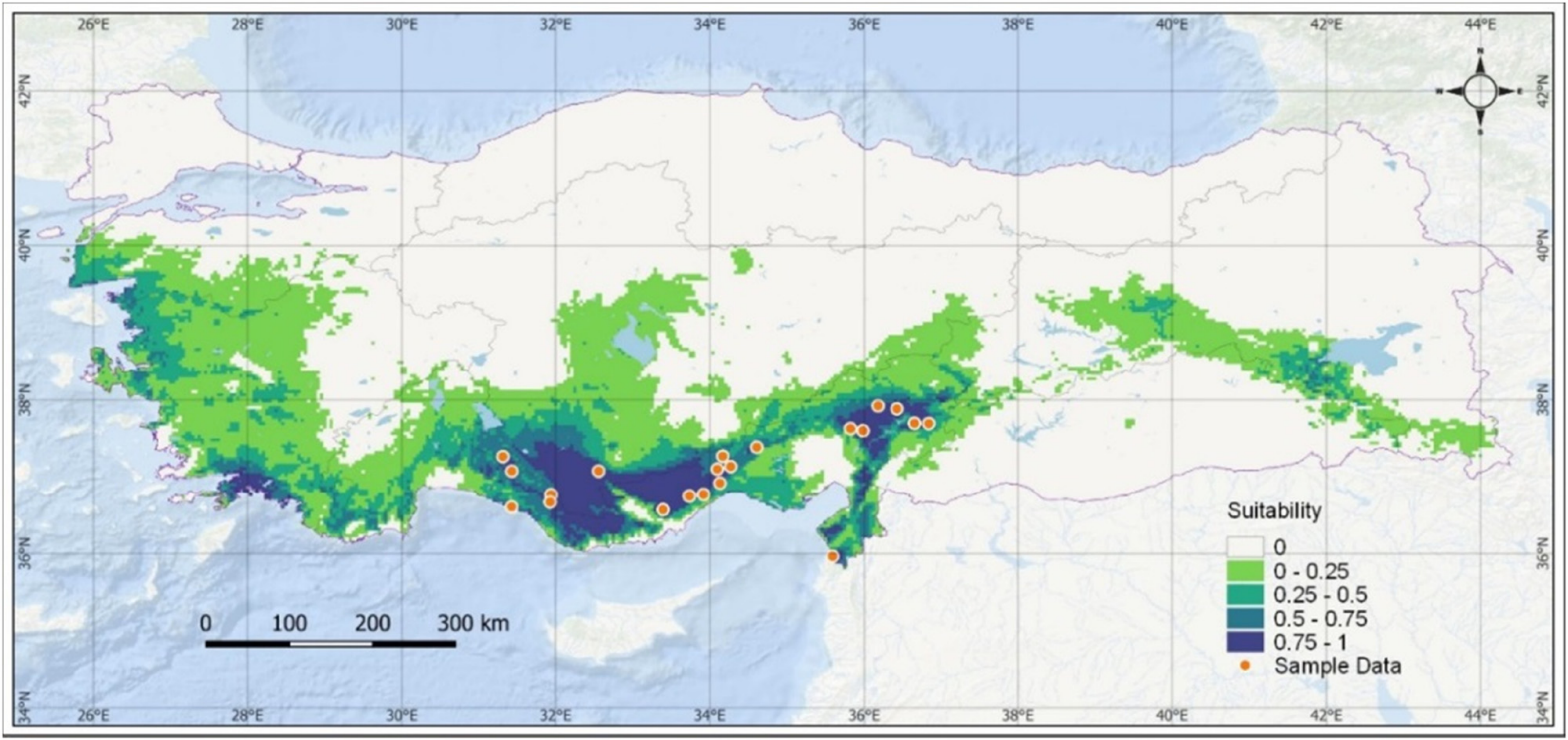
Potential current distribution of *J. drupacea*.

In the MaxEnt algorithm, the asset value occurs between 0 and 1, and the presence increases as it approaches 1. The suitability values for current and future potential spread are under five classes: “0” is not suitable, “0–0.25” is very suitable, “0.25–0.5” is less suitable, “0.5–0.75” is suitable, and “0.75–1” is very suitable. These values were collected, and the areas were calculated in km^2^ [33,34]. Comparing the potential current distribution areas of the model with the sample points indicates that they largely overlap with the natural distribution areas (Figure 5).

**Figure 6:**
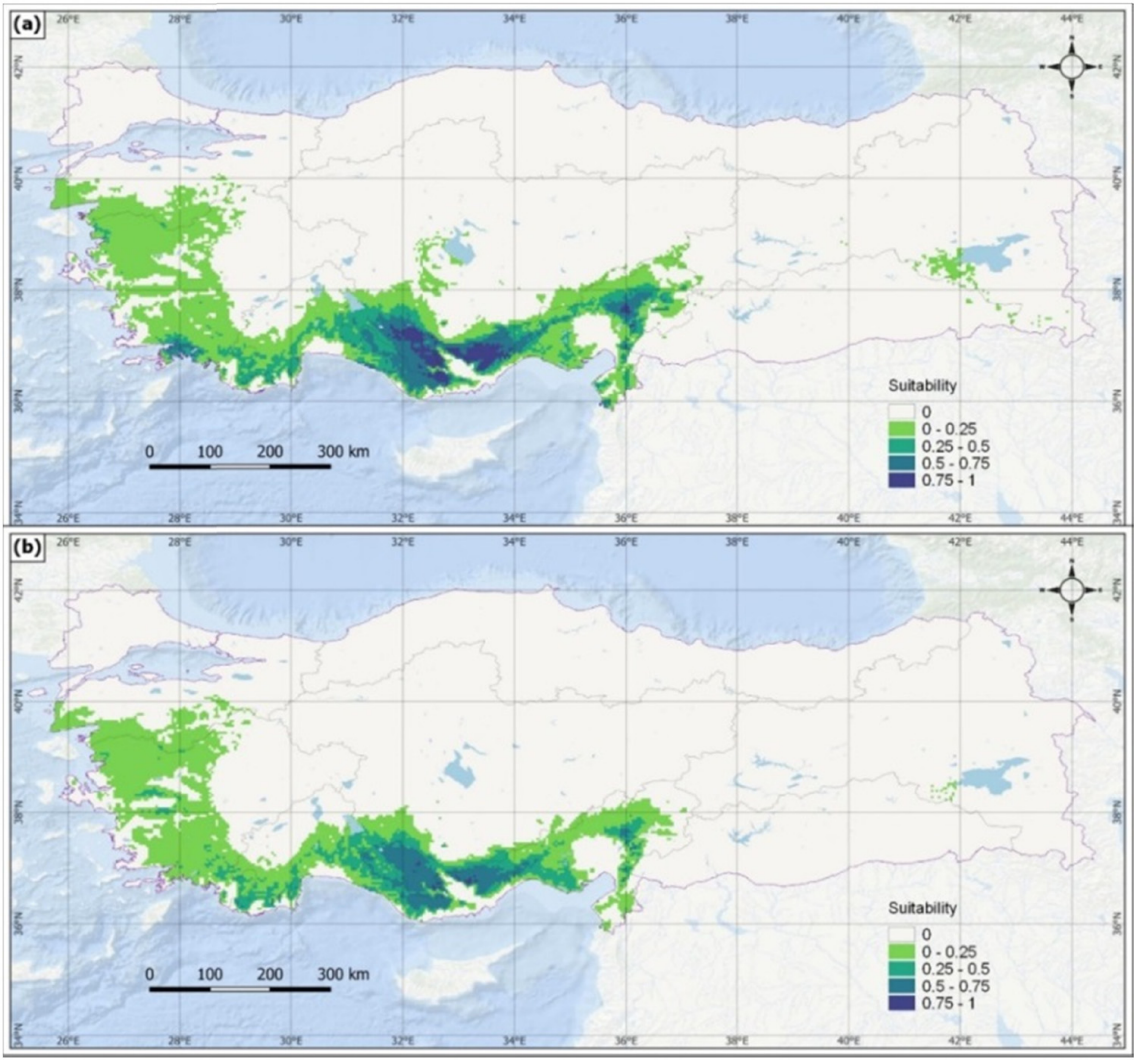
Potential distribution of *J. drupace*a for ∼2050 (a) and ∼2090 (b) according to the SSP2-4.5 scenario.

The study utilized a set of new scenarios developed for CMIP6 to offer a more comprehensive future outlook as part of the sixth Assessment Report of the Intergovernmental Panel on Climate Change (IPCC6). The IPCC AR5 incorporates four representative concentration pathways (RCPs), exploring potential future greenhouse gas emissions. These scenarios are known as RCP2.6, RCP4.5, RCP6.0, and RCP8.5. The study employed updated versions of these scenarios from CMIP6, known as shared socio-economic pathways (SSPs), which include SSP1-2.6, SSP2-4.5, SSP4-6.0, and SSP5-8.5. Notably, the study focused on the SSP2-4.5 and SSP5-8.5 scenarios. The study incorporated two specific SSPs from CMIP6: SSP2, characterized by moderate forcing levels in a temperate scenario and SSP5, representing high-level global resource usage. These scenarios, as outlined by Hausfather et al. [35], were analyzed for 2041–2060 and 2081–2100. Considering the models created according to the SSP2-45 scenario (Figure 6), there will be contractions in the species’ distribution area in the ∼2050 period, and more than half of the areas classified as very suitable and suitable will be lost. Suitable distribution areas will remain very limited and in small pieces.

**Figure 7:**
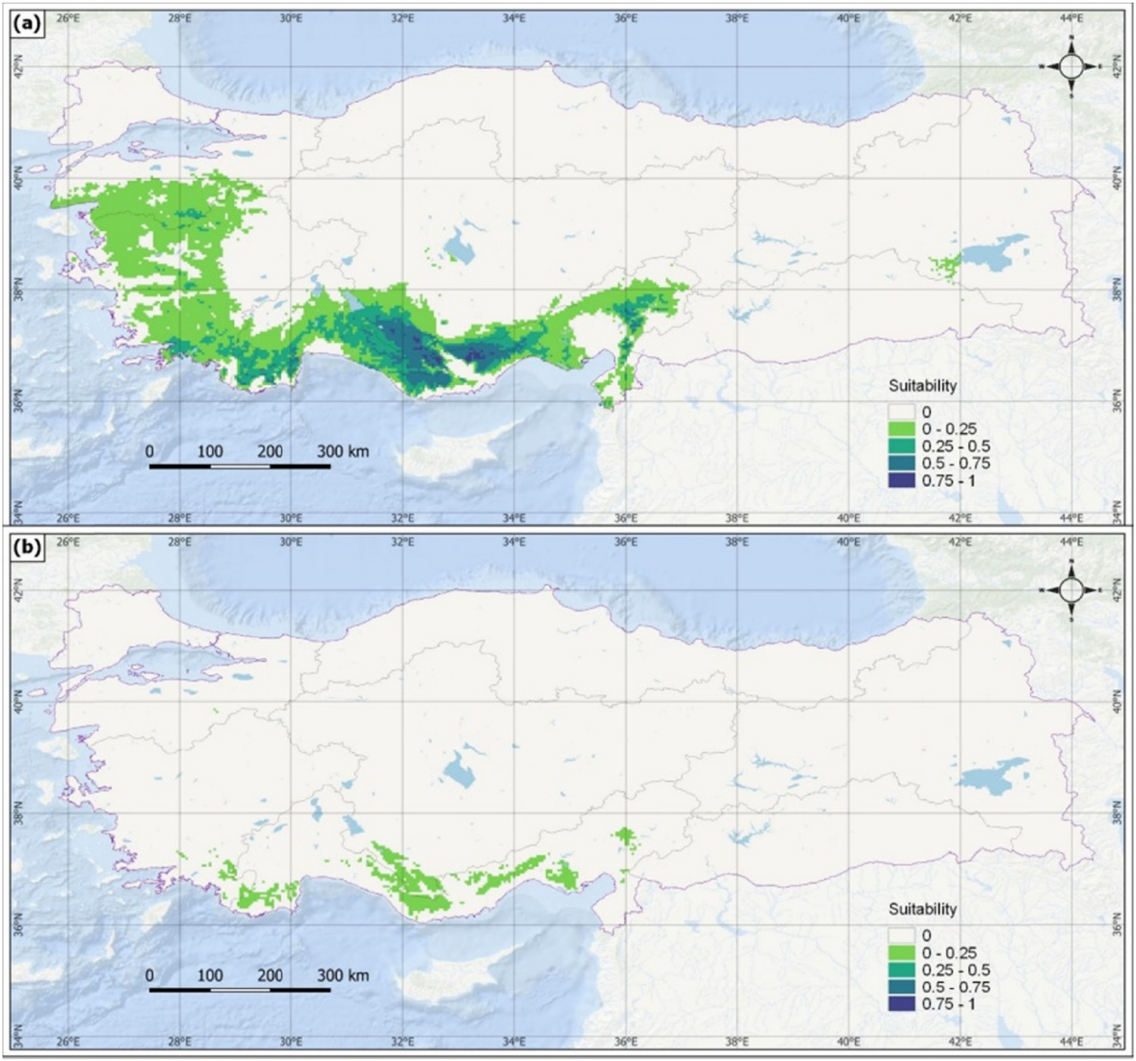
Potential distribution of *J. drupacea* for ∼2050 (a) and ∼2090 (b) according to the SSP5-8.5 scenario.

In the SSP5-85 scenario (Figure 7), very suitable distribution areas in the ∼2050 period will be limited and in small pieces, similar to the ∼2090 period in the SSP2-45 scenario, and only very few suitable areas will be available for the species distribution in the ∼2090 period (Table 4). The total of the current distribution areas indicates a decrease to 1 in 15.

**Table 4:** Potential geographical distribution of *J*. *drupacea* L. currently and in the future according to the SSP2-4.5 and SSP5-8.5 climate scenarios (km^2^)

		SSP2-4.5		SSP5-8.5	
	Current	2041–2060	2081–2100	2041–2060	2081–2100
Unsuitable	533971	636051.6	658788.3	646364.2	764438.9
Very low suitable	150677.9	97106.3	86040.8	94763.22	15506.09
Low suitable	50264.61	25002.98	22422.9	25747.09	0
Suitable	22651.89	15568.23	12314.68	12091.45	0
Very suitable	22379.7	6215.892	378.318	979.082	0

**Figure 8:**
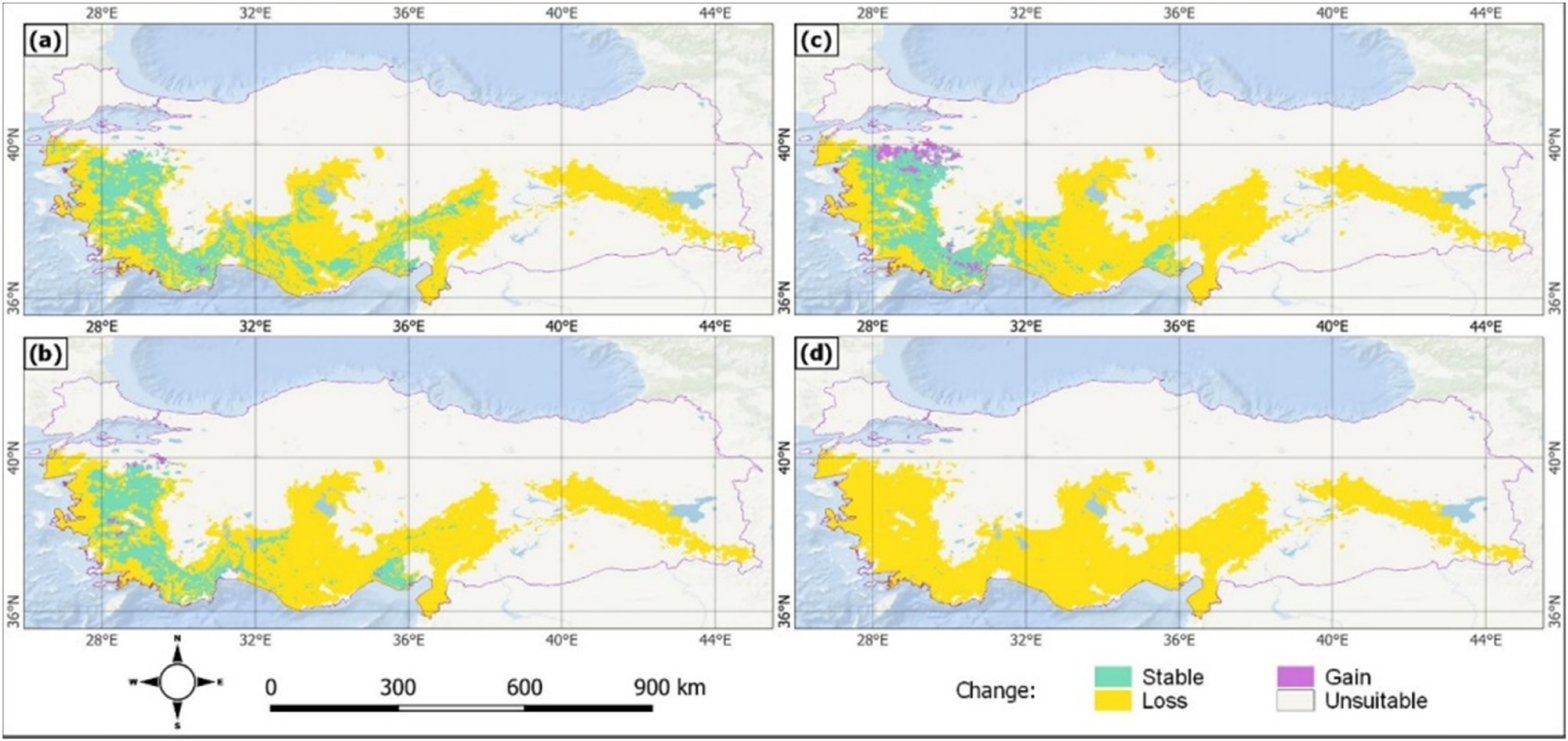
Change status of *J. drupacea* for ∼2050 and ∼2090 according to the SSP2-4.5 (a, b) and SSP5-8.5 (c, d) scenarios.

The future change of the current state of *J. drupacea* toward loss and gain is mapped in Figure 8, and the potential areas are given in Table 5 in km^2^. The maps indicate that the losses are in the majority. Only the SSP5-85 scenario for the ∼2050 period indicates a gain higher than the other period and scenario.

**Table 5:** Spatial changes between current and predicted distribution areas of *J. drupacea* under SSP2-4.5 and SSP5-8.5 scenarios for 2050 and 2090 in Türkiye (km^2^)

	SSP2-4.5		SSP5-8.5	
Change	Current to ∼2050	Current to ∼2090	Current to ∼2050	Current to ∼2090
Stable	71519.951	48118.33	55939.82	204.5
Loss	174230.408	197040.3	188098.9	245769.6
Gain	570.588	1857.909	7778.238	0
Unsuitable	533624.09	532928.5	528128.1	533971

Climate change is a critical contributor to habitat loss and biodiversity decline, as evidenced by numerous studies. The complexity of topographical variations complicates the prediction of the impact of climate change on habitat diversity and species. Nevertheless, studies indicate that certain species will likely migrate northward, while endemic species may face extinction risks [31,33,36–42]. Our results highlight the substantial influence of climate change on the Mediterranean Ecosystem, especially within the habitats of species like *J. drupacea*.

Walas et al. [43] have demonstrated that precipitation is crucial in the distribution of *J. drupacea*, mirroring our findings. Their study focused on Türkiye and examined the past, present, and future trends, predicting significant declines in the potential distribution areas of this species. In a similar study, Arslan et al. [44] determined the distribution areas of *J. foetidissima* in the 2041–2060 and 2081–2100 periods and concluded that, in the case of climate change, the adaptation resistance of the species would be low and species protection studies should be conducted.

## 4 Conclusion

“Andiz Molasses” obtained from *J. drupacea* fruits is used extensively as a folk remedy and food for its nutritional content. Our research on the ethnobotanical use of *J. drupacea* in the Antalya region determined that 98.5% of the local people use the Andiz plant, and 95.4% benefit from the obtained molasses. The molasses is used by the local people to treat colds, as an immune booster, against weakness, to treat stomach discomfort, sore throat, runny nose, and anorexia as an appetizer.

The estimated future distribution areas of *J. drupacea* due to climate change over time indicate serious contractions in the species’ distribution areas, which are currently in the Mediterranean region of Türkiye. Based on the SSP5-8.5 scenario for the ∼2090 period, very few suitable areas will remain for this species, and the continuity of the species in our country will be endangered. The tendency of the species to adapt by ascending to higher altitudes is seen in the SSP5-8.5 scenario for the ∼2050 period. However, the model results indicate a transition from an unsuitable area to very few suitable areas in this region, which will not ensure the continuity of the species. The species will suffer from serious problems, especially due to the changes in precipitation, according to the Jackknife results in the two periods.

To separate *J. drupacea* from the climate change disaster with the least damage, determining the *in situ*, *ex situ*, and gen-protection areas and conducting various studies to create populations in emerging potential areas is necessary.
